# Urinary LTE_4_ Levels as a Diagnostic Marker for IgE-Mediated Asthma in Preschool Children: A Birth Cohort Study

**DOI:** 10.1371/journal.pone.0115216

**Published:** 2014-12-18

**Authors:** Chih-Yung Chiu, Ming-Han Tsai, Tsung-Chieh Yao, Yu-Ling Tu, Man-Chin Hua, Kuo-Wei Yeh, Jing-Long Huang

**Affiliations:** 1 Department of Pediatrics, Chang Gung Memorial Hospital at Keelung, and Chang Gung University, Taoyuan, Taiwan; 2 Community Medicine Research Centre, Chang Gung Memorial Hospital, Keelung, Taiwan; 3 Division of Pediatric Pulmonology, Chang Gung Memorial Hospital and Chang Gung University, Taoyuan, Taiwan; 4 Division of Allergy, Asthma, and Rheumatology, Department of Pediatrics, Chang Gung Memorial Hospital at Linkou, and Chang Gung University, Taoyuan, Taiwan; Catholic University of the Sacred Heart, Italy

## Abstract

**Objectives:**

Leukotrienes play a central pathophysiological role in allergic asthma. The aim of this study was to investigate the utility of measuring urinary leukotriene E_4_ (LTE_4_) levels in the diagnosis of atopic diseases in early childhood.

**Methods:**

Children aged 0 through 4 years from a birth cohort in the Prediction of Allergies in Taiwanese Children (PATCH) study were enrolled. Urinary LTE_4_ levels were measured and its association between total serum IgE levels, allergen-specific IgE sensitization and atopic diseases were assessed.

**Results:**

A total of 182 children were regular followed up at clinics for a four-year follow-up period. Urinary LTE_4_ levels appeared to be elevated in children with total serum IgE levels exceeding 100 kU/L, allergen-specific IgE sensitization after 2 years of age. Elevation of urinary LTE_4_ levels (≥500 pg/mg of creatinine) significantly discriminated high serum total IgE levels (≥100 kU/L) at age 2 (*P* = 0.027). A higher level of total serum IgE or urinary LTE_4_ was significantly associated with the risk of developing allergic rhinitis and asthma at age 3. A significantly higher urinary LTE_4_ level was found in children with a combination of IgE sensitization and asthma at age 4.

**Conclusions:**

Urinary LTE_4_ levels appear to be highly associated with IgE sensitization and its related allergic airway diseases after age 2. The measurement of urinary LTE_4_ (≥500 pg/mg of creatinine) could not only be a non-invasive method for atopic predisposition but also potentially provide a strategy for the diagnosis and management of asthma in preschool children.

## Introduction

Allergic diseases are among the most common chronic diseases throughout the world and the prevalence of atopic diseases in childhood has significantly been increasing in the past few decades [1], [2]. The diagnosis of atopic diseases, especially asthma, is difficult in preschool children. The physician's diagnosis of asthma in children under the age of five is mainly based on clinical evaluation. Several studies have shown an association between prevalence of asthma and total serum immunoglobulin E (IgE) levels [3], [4]. Allergen-specific IgE antibodies also provide useful serological information in the differential diagnosis on IgE-mediated atopic diseases in young children with allergy-like symptoms [5]. Although IgE sensitization was predictive in asthma, the utility of measuring total serum IgE or allergen-specific IgE for purpose of diagnosis and management is variable. It is important to recognize that a conjunction of IgE sensitization with other biomarkers may be helpful in the diagnosis and management of early-life asthma.

Leukotrienes (LTs), including cysteinyl-LTs (LTC_4_, LTD_4_, and LTE_4_) and LTB_4_, are potent lipid mediators and they are known to play a central pathophysiological role in asthma [6]–[8]. The synthesis of cysteinyl-LTs is present in several types of inflammatory cells activated during allergic airway inflammation [9]. Elevated cysteinyl leukotriene concentrations have been detected in biological fluids, including sputum, exhaled breath condensate and bronchoalveolar lavage from patients with asthma [10]–[12]. The measurement of urinary leukotriene also provides a non-invasive method to assess changes in the rate of cysteinyl leukotriene production and excretion [13]. An increase in urinary leukotriene E_4_ (LTE_4_) levels has been reported to be correlated with the degree of airflow limitation in adults with acute asthma [14]. However, the utility of measuring urinary LTE_4_ levels in the diagnosis of asthma has not been well defined, especially in early childhood.

The aim of this study was to identify the determinants of urinary LTE_4_ levels in children aged 0 through 4 years from a birth cohort in the Prediction of Allergies in Taiwanese Children (PATCH) study. The validity of urinary LTE_4_ as a discriminative tool for IgE sensitization and atopic diseases including eczema, rhinitis and asthma was also assessed in this study.

## Methods

### Study Population

The Prediction of Allergies in Taiwanese Children (PATCH) study is a joint study initiated in 2007 to investigate the epidemiology and predictive factors of asthma and allergies in Taiwanese children, including subjects from a birth cohort and several cohorts of school and preschool children. In this birth cohort study, new born babies delivered at Chang Gung Memorial Hospital (CGMH), Keelung from October 1, 2007 to September 30, 2010 were recruited voluntarily and followed-up until the age of 4 years. Neonates born at more than 34 weeks of gestation with birth weight ≥2500 g were enrolled. Infants with any perinatal insult, significant neonatal respiratory difficulties, or congenital anomalies were excluded. Subjects who dropped out during the follow-up period were likewise excluded. This study was approved by the Ethic Committee of Chang Gung Memory Hospital (No. 102-1842C). Informed written consent was obtained from the parents of all study subjects.

### Data Collection

The parents of enrolled subjects were invited and underwent a standardized interview conducted by well-trained investigators for answering a questionnaire at birth, 6 months and at 1, 2, 3 and 4 years of follow-up. The questionnaire was derived from the well-validated International Study of Asthma and Allergies in Childhood (ISAAC) questionnaire [15]. The details of information regarding demographic data, family atopy history, general health information, and questions on clinical symptoms and diagnosis of atopic diseases were collected.

### Evaluation and Diagnosis of Atopic Diseases

Specific questions related to the development of allergic/atopic diseases and symptoms were regularly inquired and evaluated by a pediatric pulmonologist at outpatient clinics. Eczema was diagnosed as a pruritic rash over the face and/or extensors with a chronic relapsing course as described by Hanifin and Rajka [16]. Rhinitis was diagnosed as ever having the symptoms representing rhinitis such as sneezing, nasal congestion, itching, rhinorrhea in the last 12 months or current use of medication for these symptoms [17]. Asthma was diagnosed as ever having asthma with the occurrence of recurrent wheeze in the last 12 months or current use of asthma medication. Early-onset asthma was defined as asthma beginning before the age of 2 [18].

### Total and Allergen-Specific Serum Immunoglobulin E

Serum samples were collected and measured at 6 months, and 1, 1.5, 2, 3 and 4 years of age. The serum level of total immunoglobulin (Ig) E was measured by ImmunoCAP (Phadia, Uppsala, Sweden). Allergen-specific IgE was determined by a commercial assay for IgE (ImmunoCAP Phadiatop Infant; Phadia) and a mix of three most common food allergens (egg white, milk and wheat) and three most common inhalant allergens (*D. pteronyssinus*, *D. farina* and *C. herbarum*) were measured. The cut-off values for each ImmunoCAP Phadiatop Infant class 0, 1, 2, 3 and >3 are 0, 0.35, 0.7, 3.5 and ≥17.5 kU/L, respectively. Values of a total IgE level exceeding 100 kU/L or ImmunoCAP Phadiatop Infant of ≥0.35 kU/L (≥ class 1) were considered indicative of IgE sensitization [3], [19].

### Urinary Leukotriene E_4_ Measurements

Spot urine samples were collected and measured at 6 months, and 1, 1.5, 2, 3 and 4 years of age for the changes of LTE_4_ levels. Urine samples were stored at −80 degree Celsius in aliquots until required. For each use, an aliquot was thawed, used and any remnants discarded after completion of the experiment. LTE_4_ in urine was measured by ACE Enzyme Immunoassay Kit (Cayman Chemical, Ann Arbor, MI, USA), according to the manufacturer's instructions. This assay was based on the competition between free LTE_4_ and a LTE_4_ tracer (LTE_4_-acetylcholinesterase conjugate) for a limited amount of LTE_4_ antiserum. The detection limit in the assay was <8 pg/mL. This assay has shown excellent precision (intra-assay and inter-assay coefficient of variation <10%) [20]. Urinary LTE_4_ levels were reported in picograms (pg) per milliliter and standardized per milligram (mg) of creatinine (measured by Jaffee methodology) in order to control for urine volume. Urinary LTE_4_ concentrations were therefore reported as pg/mg of creatinine.

### Statistical Analysis

Demographic data of population characteristics obtained by questionnaire and the prevalence of physician-diagnosed atopic diseases were collected and analysed. The Student *t* test was used to compare continuous variables, and the differences between continuous variables with non-normal distribution were estimated with the Mann-Whitney test. Urinary LTE_4_ cut-off levels with the highest accuracy were determined by receiver operating characteristic (ROC) curves. The associations of atopic diseases measured as a binary outcome with total serum IgE levels and urinary LTE_4_ levels were calculated by the odds ratio (OR), using standard methods of logistic regression analysis. Statistical analysis was performed by using the Statistical Program for Social Sciences (IBM SPSS Statistics for Windows, Version 20.0. Armonk, NY: IBM) and graphs were drawn using GraphPad Prism Version 5.01 software (GraphPad Software Inc, California, USA). All statistical hypothesis tests were two tailed and a *P* value of less than 0.05 was considered to be significant.

## Results

### Population Characteristics

**Figure 1:**
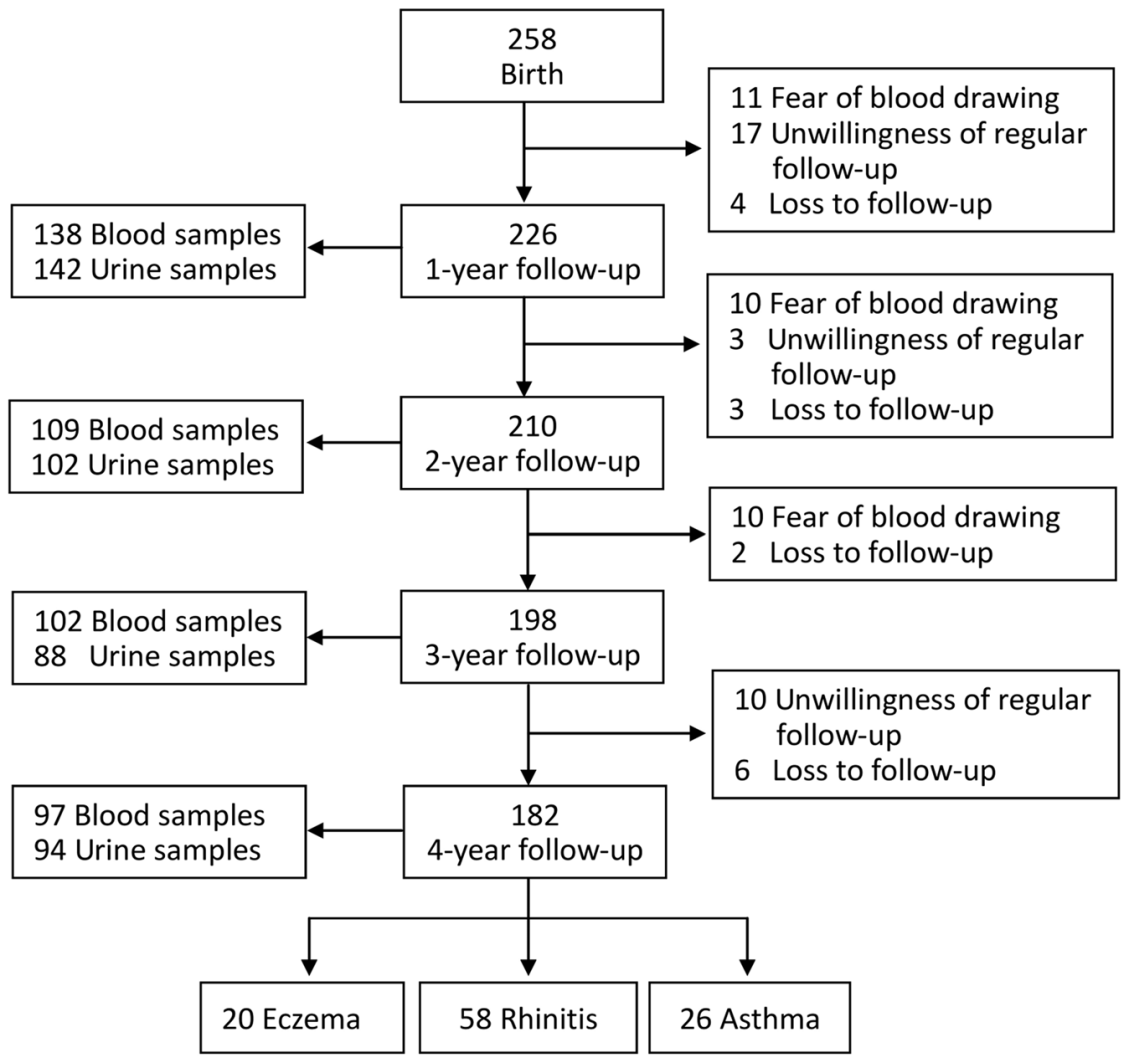
Schematic presentation of the recruitment process of the study subjects.

A total of 258 children who fulfilled the inclusion criteria were voluntarily enrolled; 226 (87.6%), 210 (81.4%), 198 (76.7%) and 182 (70.5%) children were regular followed up at clinics for a one, two, three and four year follow-up respectively. The major reasons for dropout were the fear of blood drawing and parents' unwillingness of regular outpatient follow-up. Atopic diseases including eczema, rhinitis and asthma were diagnosed in 20, 58 and 26 children, respectively, at 4 years of age (Fig. 1).

### Prevalence of Sensitization to Food and Inhalant Allergens

**Figure 2:**
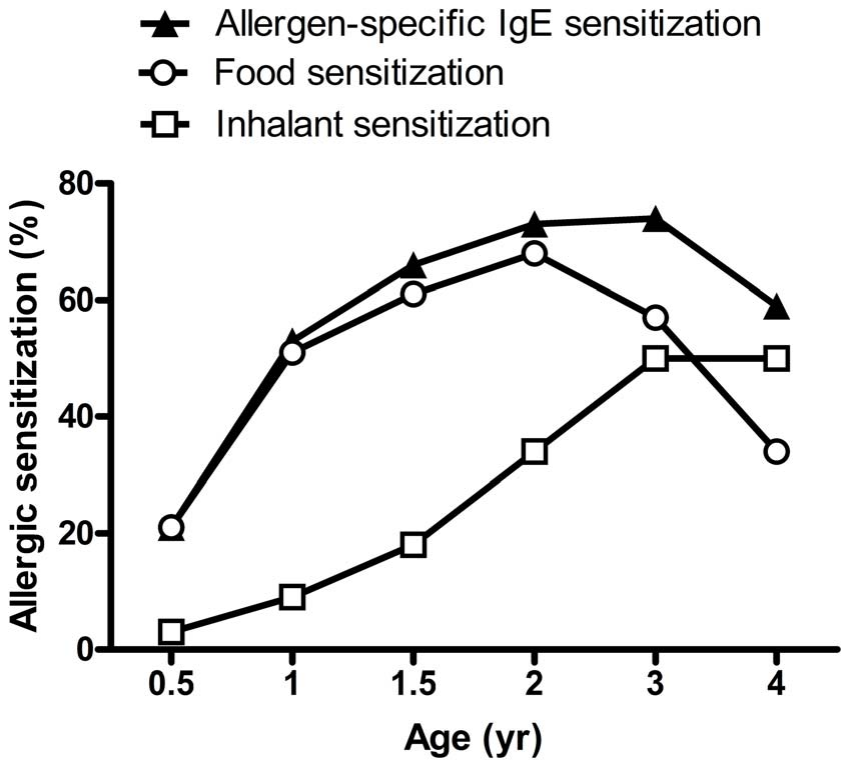
Sensitization patterns to food and inhalant allergens from age 0 to age 4.

Serum total IgE levels and allergen-specific IgE for food and inhalant allergens was measured at 6 months, and 1, 1.5, 2, 3 and 4 years of age during follow-up. Sensitization patterns to food and inhalant allergens are shown in Fig. 2. The prevalence of allergen-specific IgE sensitization showed to be increased gradually with increasing age, from 21% at 6-month-old to 74% at 3-year-old. The prevalence of food allergen sensitization increased markedly after 6 months of age and reached up to 68% at 2 years of age. In contrast, the prevalence of sensitization to inhalant allergens was only 9% at age 1, but increased markedly after 2 years of age. At 4 years of age, the prevalence of sensitization to food allergens declined significantly to 34% but there was a considerable increase in the prevalence of sensitization to inhalant allergens up to 50%.

### Changes of Urinary LTE4 Levels Categorized by High Serum IgE Levels and Allergen-specific IgE Sensitization

**Figure 3:**
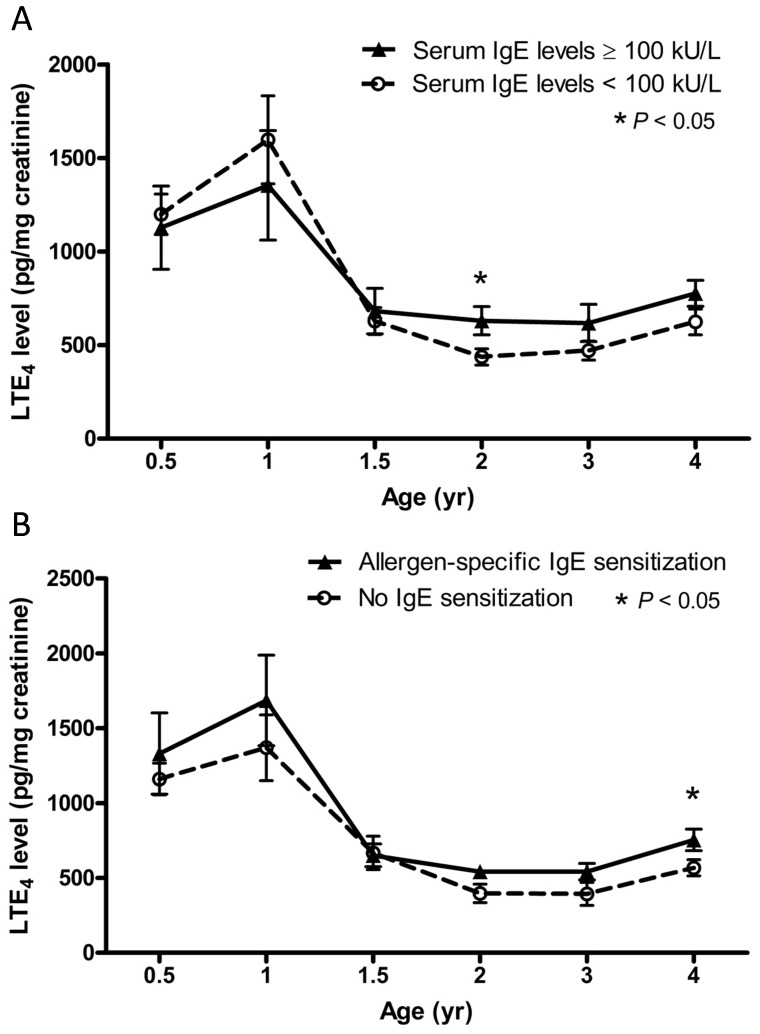
Levels of urinary LTE_4_ levels categorized by high serum IgE levels (A) and allergen-specific IgE sensitization (B) at different years of age. Data shown are mean ± SEM. *P<0.05 as compared to children without IgE sensitization.

Urinary LTE4 levels were measured at 6 months, and 1, 1.5, 2, 3 and 4 years of age during follow-up. Fig. 3 shows the changes of urinary LTE4 levels categorized by high serum IgE levels (Fig. 3A) and allergen-specific IgE sensitization (Fig. 3B) at different years of age. Urinary LTE4 concentrations declined significantly after age 1 and reduced slightly after the age of 1.5. In children with serum IgE level above 100 kU/L or allergen-specific IgE sensitization, there was a relatively high level of urinary LTE4 after 1.5 years of age in comparison with children without IgE sensitization. Furthermore, a significantly higher level of urinary LTE4 was found in children with high serum total IgE levels (≥100 kU/L) at age 2 and in children with food, inhalant or allergen-specific IgE sensitization at age 4.

### Association between Total Serum IgE Levels, Urinary LTE4 Levels and Atopic Diseases

**Figure 4:**
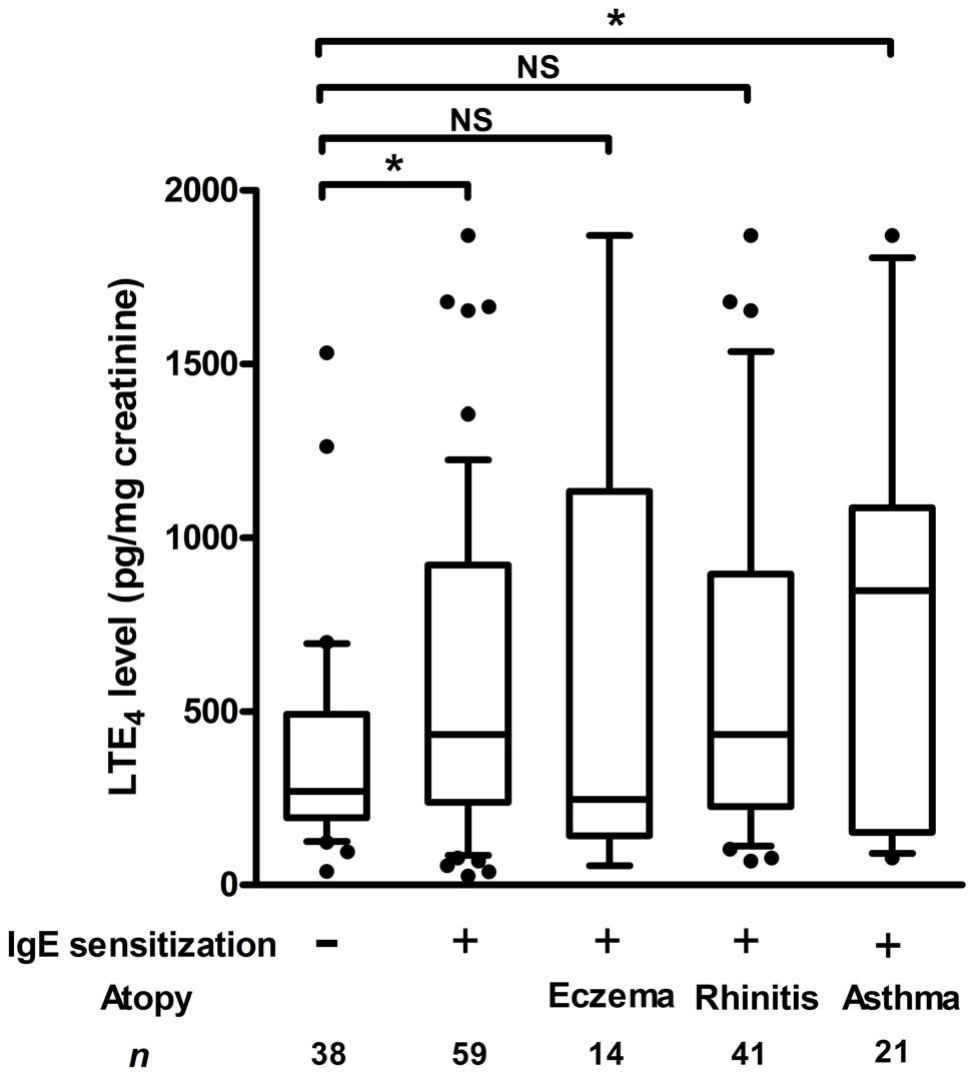
Box plots showing median and interquartile ranges of urinary LTE_4_ levels at age 4 by subject groups. Dots beyond the bounds of the whiskers denote outliers. *P* values refer to the comparisons indicated by the marker. *P<0.05; NS, not statistically significant.

A ROC curve was generated to determine the sensitivity and specificity of urinary LTE4 levels for discriminating children with and without high serum total IgE levels. The urinary LTE4 levels had the highest area under the ROC curve (AROC) significantly different from 0.5 at 2 years of age (AROC  = 0.66; 95% confidence interval: 0.51–0.77; P = 0.027). The highest combination of sensitivity and specificity was observed with a cut-off level of 500 pg/mg of creatinine (61.3% and 70.5%, respectively) for predicting high serum total IgE levels. The relationships between high serum total IgE levels (≥100 kU/L), high urinary LTE4 levels (≥500 pg/mg of creatinine) and the risk of atopic diseases during early childhood are shown in Table 1. A higher level of total serum IgE and urinary LTE4 was significantly associated with the risk of allergic rhinitis and asthma at age 3. Although there was no significant association between urinary LTE4 levels and atopic diseases at age 4, a significantly higher LTE4 level was found in children with a combination of IgE sensitization and asthma. Urinary LTE4 levels in the combined analyses of IgE sensitization and atopic diseases diagnosed at age 4 is shown in Fig. 4.

**Table 1:** Relationships between total serum IgE levels, urinary LTE_4_ levels and risk of atopic diseases.

Age	Outcome	Serum IgE ≥100 (kU/L)			Urinary LTE_4_ ≥500 (pg/mg of creatinine)		
		OR	95% CI	*P* value	OR	95% CI	*P* value
2Y	Eczema	1.42	0.48–4.21	0.531	1.24	0.38–4.08	0.721
	Allergic rhinitis	3.90	1.60–9.54	0.003	2.22	0.89–5.52	0.088
	Early-onset asthma	2.27	0.82–6.29	0.116	1.78	0.64–4.98	0.274
3Y	Eczema	2.02	0.71–5.75	0.189	1.47	0.49–4.43	0.494
	Allergic rhinitis	3.87	1.52–9.87	0.005	3.33	1.20–9.23	0.021
	Asthma	5.40	1.99–14.62	0.001	2.98	1.09–8.18	0.034
4Y	Eczema	0.63	0.18–2.21	0.475	0.49	0.14–1.71	0.264
	Allergic rhinitis	4.29	1.60–11.49	0.004	2.00	0.82–4.86	0.126
	Asthma	5.41	1.91–15.36	0.001	1.19	0.45–3.17	0.729

IgE, immunoglobulin E; LTE_4_, leukotriene E_4_; OR, odds ratio; CI, confidence interval.

## Discussion

In young children, the diagnosis of atopic diseases must be based largely on clinical judgment, and should be periodically reviewed as the child grows. Clinically, the prevalence of allergic respiratory diseases increases with increasing age and a rapid upward trend is observed after 2 year of age [21]. It is also known that most school-aged asthmatic children have a history of airway obstruction during the first 2 to 3 years of life, at which age asthma might be in the course of evolving from infection related to allergy predominantly related [22], [23]. These findings highlight the importance that a prompt and correct diagnosis of atopic diseases, particularly asthma, in young children may allow for better management and, potentially, for reduced morbidity and mortality.

IgE is a critical component of allergic diseases. IgE binds to allergens and triggers the release of substances from mast cells that can cause inflammation. Total serum IgE is an original screening test for atopic predisposition and a total IgE level exceeding 100 kU/L is highly suggestive of allergy [24], [25]. However, some children with significant allergy problems can have normal or moderately elevated IgE levels. Sensitization to allergens has also been recognized as the most important risk factor for atopic diseases. A moderate amount of specific IgE to a particular allergen may have much greater significance for a relatively lower total IgE levels [19]. Although an elevated IgE level is associated with an increased risk of atopic disease as in this study, IgE sensitization may only provide a useful context for assessing the likely significance of atopic diseases, rather than specific information for the diagnosis or management of asthma [3], [4], [24].

Leukotrienes have an established role in a wide variety of inflammatory diseases, including atherosclerotic cardiovascular disease, inflammatory bowel disease, and atopic diseases such as allergic rhinitis and asthma [26]. LTE_4_ can be regarded as the end product of cysteinyl-LTs and used as an appropriate marker for monitoring the systemic production of cysteinyl-LTs [27], [28]. Although urine has been found to be the most suitable biological fluid for measuring the whole body production of cysteinyl-LTs, the levels of urinary LTE_4_ may heavily vary depending on age [29]. In this study, urinary LTE_4_ levels have shown to decrease progressively with increasing age during early childhood. It must be emphasized that a strict assessment of the normal reference values of urinary LTE_4_ based on the subjects' age is considered a crucial step to investigate the role of urinary LTE_4_ levels in young children with atopic diseases.

In atopic diseases, Th_2_ cells control the regulation of B cell class-switching to IgE, which induces mast-cell activation and the development of allergic reaction [30]. In this study, compared with children without IgE sensitization, urinary LTE_4_ levels appeared to be significantly elevated in children with IgE sensitization after 2 years of age, which is the age at which the prevalence of allergic respiratory diseases increases markedly. In addition, an elevated urinary LTE_4_ level has shown to be not only associated with IgE sensitization but also IgE-mediated asthma in this study. These findings indicate the pivotal role of LTs in the complex network of IgE-mediated immune responses that characterizes allergic airway diseases. These results also support the previous reports that LTs may regulate Th_2_ cell-dependent inflammatory response, in which simultaneous activation of mast cells through IgE leads to allergic airway inflammation [31], [32].

Leukotrienes play a central pathophysiological role in allergic rhinitis and asthma [8], [9]. Leukotriene modifiers have been approved to have a therapeutic role in severe asthma as they improve pulmonary function, and reduce acute asthma exacerbations, and the required dose of inhaled corticosteroids [32]–[35]. In this study, serum IgE levels provide a screening test to distinguish atopic and non-atopic children. In contrast, the elevation of urinary LTE_4_ level after age 2 was consistent with the increase in the risk of high serum total IgE levels, while a concentration greater than 500 pg/mg creatinine may shift the odds to make the diagnosis of asthma other than only the prediction of atopy in preschool children. The measurement of urinary LTE_4_ may therefore not only provide a simple non-invasive method for predicting atopy but also a complementary strategy for the diagnosis and management of asthma.

Limitations of this study include a relatively small sample size of only 182 case subjects and a limited power to detect a statistically significant association for subanalyses. There is also a limitation on the interpretation of our findings, because without correction for multiple comparisons. A 4-year follow-up in this birth cohort study may not predict if children with episodic viral wheeze will develop asthma in later years. The strength of the present study however lies in its longitudinal design, allowing sequential and concurrent measurements of IgE sensitization, urinary LTE_4_ levels and the accurate diagnostic evaluations for atopic diseases at outpatient clinics. Although a larger and longer study may be needed to confirm the findings presented in this study, this birth cohort study has demonstrated the importance of urinary LTE_4_ in predicting atopy and diagnosing asthma in preschool children.

In conclusion, the IgE sensitization is a measure that can inform clinicians about potential and risk of atopic diseases in young children. Urinary LTE_4_ levels appear to be highly associated with IgE sensitization and its related allergic airway diseases after age 2. The ability of urinary LTE_4_ levels in discriminating high serum total IgE levels provides a simple non-invasive method for predicting atopic diseases in young children. An elevated urinary LTE_4_ level (≥500 pg/mg of creatinine) appears to provide more specificity in diagnosis of IgE-mediated asthma. In preschool children, the measurement of urinary LTE_4_ could not only contribute to identify atopic predisposition but also potentially provide a strategy for the diagnosis and management of asthma.
